# The personality trait of behavioral inhibition modulates perceptions of moral character and performance during the trust game: behavioral results and computational modeling

**DOI:** 10.7717/peerj.1631

**Published:** 2016-02-08

**Authors:** Milen L. Radell, Rosanna Sanchez, Noah Weinflash, Catherine E. Myers

**Affiliations:** 1 Neurobehavioral Research Laboratory, VA New Jersey Health Care System, East Orange, NJ, United States; 2 Department of Psychology, Rutgers, The State University of New Jersey—Newark, Newark, NJ, United States; 3 Honors College, Rutgers, The State University of New Jersey—Newark, Newark, NJ, United States; 4 Department of Pharmacology, Physiology & Neuroscience, New Jersey Medical School, Rutgers, The State University of New Jersey, Newark, NJ, United States

**Keywords:** Decision making, Behavioral inhibition, Trustworthiness, Computational model, Trust game, Reinforcement learning

## Abstract

Decisions based on trust are critical for human social interaction. We judge the trustworthiness of partners in social interactions based on a number of partner characteristics as well as experiences with those partners. These decisions are also influenced by personality. The current study examined how the personality trait of behavioral inhibition, which involves the tendency to avoid or withdraw from novelty in both social and non-social situations, is related to explicit ratings of trustworthiness as well as decisions made in the trust game. In the game, healthy young adults interacted with three fictional partners who were portrayed as trustworthy, untrustworthy or neutral through biographical information. Participants could choose to keep $1 or send $3 of virtual money to a partner. The partner could then choose to send $1.5 back to the participant or to keep the entire amount. On any trial in which the participant chose to send, the partner always reciprocated with 50% probability, irrespective of how that partner was portrayed in the biography. Behavioral inhibition was assessed through a self-report questionnaire. Finally, a reinforcement learning computational model was fit to the behavior of each participant. Self-reported ratings of trust confirmed that all participants, irrespective of behavioral inhibition, perceived differences in the moral character of the three partners (trustworthiness of good > neutral > bad partner). Decisions made in the game showed that inhibited participants tended to trust the neutral partner less than uninhibited participants. In contrast, this was not reflected in the ratings of the neutral partner (either pre- or post-game), indicating a dissociation between ratings of trustworthiness and decisions made by inhibited participants. Computational modeling showed that this was due to lower initial trust of the neutral partner rather than a higher learning rate associated with loss, suggesting an implicit bias against the neutral partner. Overall, the results suggest inhibited individuals may be predisposed to interpret neutral or ambiguous information more negatively which could, at least in part, account for the tendency to avoid unfamiliar people characteristic of behaviorally inhibited temperament, as well as its relationship to anxiety disorders.

The trust game is a paradigm widely used to study how humans modulate behavior based on social interactions (Rekosh & Feigenbaum, 1966; Berg, Dickhaut & McCabe, 1995; King-Casas et al., 2005; Delgado, Frank & Phelps, 2005; Fareri, Chang & Delgado, 2012; Fouragnan et al., 2013). In the game, on each trial, the participant receives an endowment (e.g., $1) and can choose to either keep the money or share it with a fictional partner. If the money is shared, the partner receives a larger sum (e.g., $3) which he in turn can choose to keep or split evenly with the subject. Participants can maximize winnings by learning to share with (trust) a partner who reliably shares in return, but keep the endowment from a partner who has violated trust in the past. In one variant of the trust game (Delgado, Frank & Phelps, 2005), subjects view fictional biographies of several partners, who are portrayed as morally trustworthy, untrustworthy, or neutral. This prior declarative information skews subsequent behavior so that subjects tend to share more with the partner who is perceived as morally trustworthy, and less with the partner viewed as untrustworthy, even if both partners actually reciprocate at the same level during the game. This suggests that prior moral information modulates trial-and-error learning from experience. The type of prior information is also related to the neural substrates involved in the task. For example, greater activation in the caudate nucleus was observed when there was no prior information, or when this information was neutral, relative to when this information suggested the partner is trustworthy (Delgado, Frank & Phelps, 2005). In a modified version of the trust game, Fouragnan et al. (2013) also found greater activation in the caudate when no prior information was available.

Reinforcement learning models have been used to understand these processes (e.g., Fareri, Chang & Delgado, 2012; Fouragnan et al., 2013). These models are a type of computational model that learns to execute responses that maximize expected future reward, and typically contain a number of free parameters such as learning rate and tendency to explore new vs. execute previously-reinforced responses (Barto, Sutton & Anderson, 1983; Sutton, 1988; Watkins & Dayan, 1992). Using mathematical estimation techniques, a set of parameter values is derived for each participant so that model performance best mimics the participant’s actual value on a trial-by-trial basis. In many cases, subject performance is best fit when distinct parameters are allowed to encode learning from reward vs. learning from punishment (e.g., Frank et al., 2007; Fareri, Chang & Delgado, 2012). In the trust game, fit may be further improved when additional free parameters are allowed to encode the “biases” engendered by prior information (Fareri, Chang & Delgado, 2012; Fouragnan et al., 2013), when learning rates are allowed to differ for different partners (Fareri, Chang & Delgado, 2012) or when the degree of expected “trustworthiness” for each partner can vary across trials as a result of experience (Chang et al., 2010). Thus, although there are commonalities across subjects, putatively healthy individuals vary in how strongly they evidence this effect. Currently, there is incomplete understanding of what underlying biological or mental processes produce this variability.

One factor that may modulate individual differences in performance on the trust game is personality. Here, we focus on the trait of behavioral inhibition (BI), which is defined as a temperamental tendency to avoid or withdraw from novelty in social and non-social situations (Gladstone & Parker, 2005). Individuals who score high in BI have increased risk for developing anxiety disorders (Rosenbaum et al., 1993; Turner, Beidel & Wolff, 1996; Mick & Telch, 1998; Clauss & Blackford, 2012; Clauss, Avery & Blackford, 2015) and tend to be less trusting of others (Oskarsson et al., 2012). As such, our initial hypothesis was that high-BI individuals will choose to keep more (rather than share) in the game, indicating lower overall trust. Since previous studies have shown that the type of prior information about the partner is important (Delgado, Frank & Phelps, 2005; Fouragnan et al., 2013), we predicted that how BI modulates trust will also depend on this factor. Finally, recent studies also suggest that high-BI individuals show accelerated associative learning, including eyeblink classical conditioning (Caulfield, McAuley & Servatius, 2013; Holloway et al., 2014; Caulfield, VanMeenen & Servatius, 2015) and computer-based operant tasks (Sheynin et al., 2013; Sheynin et al., 2014). The latter studies have been interpreted as suggesting that high-BI individuals have a bias for stimulus–response learning, relative to low-BI peers. As such, we also predicted that although high-BI individuals might initially be less trusting, they would also adjust those beliefs faster than low-BI peers throughout the game, due increased ability to update response rules based on trial-and-error learning.

In the current study, a large sample of undergraduate students were administered the trust game as well as a questionnaire assessing BI. First, we examined whether BI modulated evaluations of trust, assessed before and after the game, and whether it modulated performance during the trust game itself. Specifically, we predicted that differences in BI may lead to differences in the initial estimate of trustworthiness for each partner, as well as in how individuals learn over the course of the game. As above, since previous studies have shown that the type of prior information about the partner is important (Delgado, Frank & Phelps, 2005; Fouragnan et al., 2013), we predicted that these differences will also be a function of the prior beliefs about the partner.

Second, we applied a series of RL models previously used to assess performance on this game to derive estimated parameters for each participant, and asked whether the derived parameter values were significantly different for high-BI vs. low-BI participants. If such differences exist, they might help elucidate the mechanisms by which BI affects choice behavior and its modulation by prior social information.

## Methods

### Experiment

#### Participants

A total of 114 participants (73 female, mean age 21.4 years, SD 5.0 years) were recruited from the Psychology Department in Rutgers University-Newark. Each received research credit in a psychology class for participation in a one-hour session. Of the sample, 28 self-reported race as White/Caucasian, 20 as Black/African-American, 19 as Asian, 2 as Pacific Islander, 1 as Native American, and the remainder (44) as Other or Mixed-Race; 29 self-reported ethnicity as Hispanic.

All participants provided written informed consent before initiation of any behavioral testing. Procedures were approved by the Rutgers University Institutional Review Board (protocol number 14-087M). This protocol was approval on August 18, 2013 and has received annual continuing review thereafter. The procedures also conformed to guidelines established by the Federal government and the Declaration of Helsinki for the protection of human subjects.

#### Procedure

Testing took place in a quiet room, with the participant seated at a comfortable viewing distance from the computer screen. Participants completed two short paper-and-pencil questionnaires: a demographic questionnaire that asked for age, education, racial, and ethnic information (summarized above), as well as the Adult Measure of Behavioural Inhibition (AMBI; Gladstone & Parker, 2005). The AMBI assesses current (adult) BI via 16 questions relating to tendency to respond to new stimuli with inhibition and/or avoidance. Scores on this scale range from 0 to 32, with higher scores indicating greater BI. Participants who scored lower than 16 on the AMBI were classed as “uninhibited,” with the remainder classed as “inhibited,” a cutoff used by the authors of the scale (Gladstone & Parker, 2005) as well as prior studies comparing BI to behavior and to symptomatology (e.g., Myers et al., 2013; Sheynin et al., 2013; Caulfield, VanMeenen & Servatius, 2015).

**Figure 1:**
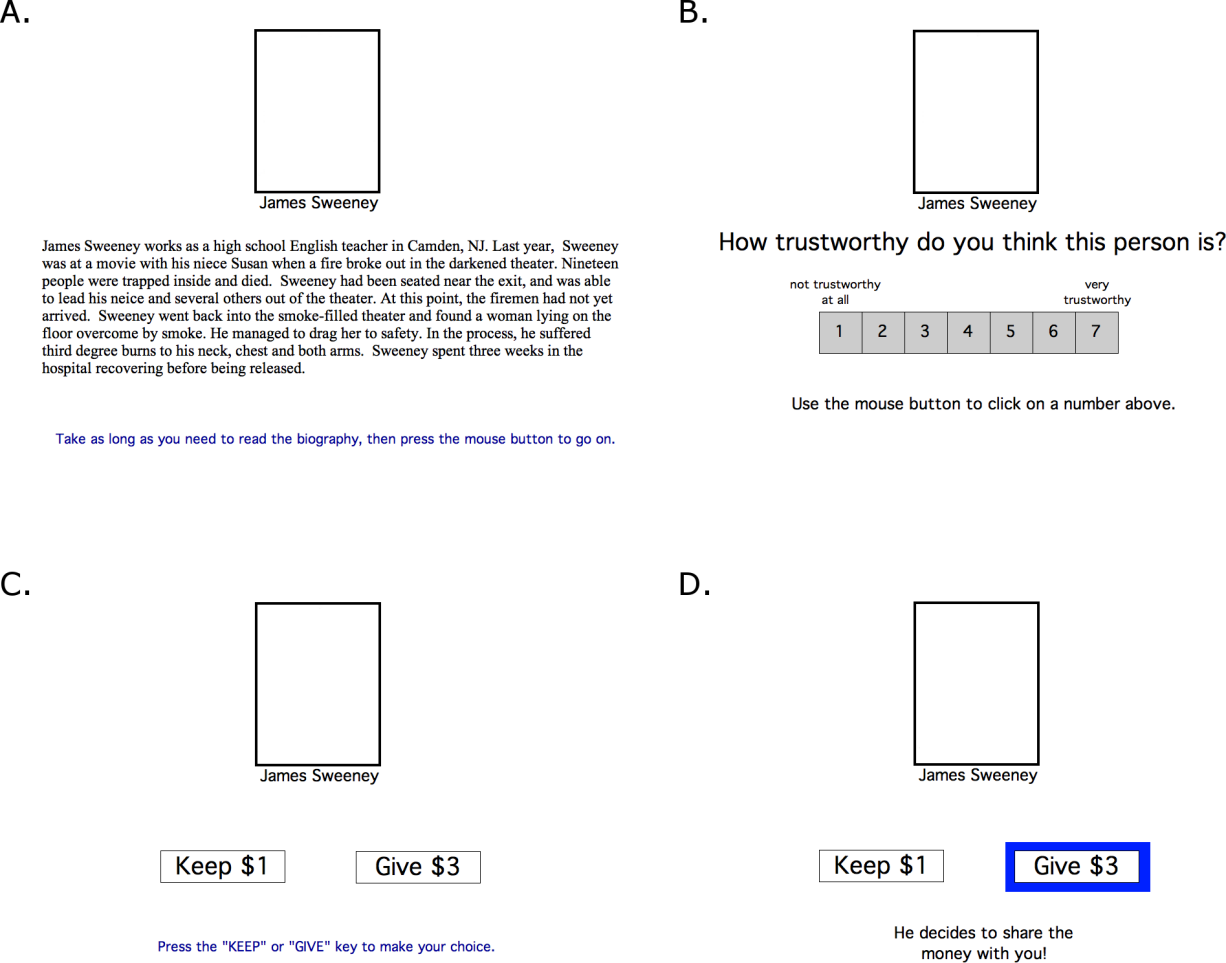
Screen events in the trust game. (A) At the start of the experiment, subjects were shown the names and faces of each of three fictional partners, along with a short biography of each. A photo of the partner appeared in the white rectangle above the name. (B) After reading all three biographies, participants were shown each partner and asked to rate his trustworthiness on a 7-point scale. (C) On each trial in the game, a partner was shown and the participant could choose to keep $1 or give $3 to the partner. (D) If the participant chose to share, the partner could either share the money back (in which case the participant received $1.50), or keep all the money (in which case the participant received $0).

Participants then received a version of the trust game modified from Delgado, Frank & Phelps (2005). This was presented on a Macintosh iBook or equivalent computer, programmed in the SuperCard programming language (Allegiant Technologies, San Diego, CA). The keyboard was masked except for two keys labeled “KEEP” and “SHARE” used to enter responses. First, each participant saw the photo and name of three fictional male partners, each with a short biography (Fig. 1A). Faces and names were the same as in Delgado, Frank & Phelps (2005), with faces originally selected from white male faces rated as neutral in the NimStim stimulus set (Tottenham et al., 2002; also see Tottenham et al., 2009). However, the biographies were simplified (see Appendix). As in the prior study, the biographies portrayed each of the partners as morally trustworthy (“good partner”), untrustworthy (“bad partner”), or neutral (“neutral partner”). Participants were not told anything about how each partner might play, including whether behavior of the partners would correspond to information in the biography. Assignment of names and faces to biographies was counterbalanced across subjects. Participants viewed the biographies on the screen for as long as they wished; after viewing all three, they were then shown each partner (name and face) and asked to rate their trustworthiness on a scale from 1 (completely untrustworthy) to 7 (completely trustworthy) (Fig. 1B).

Next the trust game began. On each trial, subjects saw the photo and name of one of the three partners, and were given a choice of keeping $1 or sharing $3 with that partner (Fig. 1C). If the money was shared, the partner could in turn choose to keep it all (in which case the participant received $0) or to reciprocate (in which case the participant received $1.50 (Fig. 1D). On any trial in which the participant chose to share, the partner always reciprocated with 50% probability, irrespective of how that partner was portrayed by the biography. The game included 72 trials, 24 with each partner (in pseudorandom order, but with the constraint that each partner appeared once every 3 trials).

Following completion of the trust game, subjects were asked to re-rate each partner for trustworthiness using the seven-point scale (Fig. 1B).

#### Data analysis

Linear regression was used to assess whether score on the AMBI could predict trust ratings and behavior in the game for each partner. When appropriate, analysis of variance (ANOVA) was used as a secondary test with BI class (uninhibited or inhibited) entered as a factor. Levene’s test and Mauchly’s test were used to confirm equality of variance and covariance; where Mauchly’s test was failed (*p* < .05), Greenhouse-Geisser correction was used to adjust degrees of freedom for interpreting *p*-values from *F*-values. Where multiple comparisons were made, Bonferroni correction was used to protect against accumulating risk of Type I error. Adjusted values for alpha are only shown where the obtained *p*-value is less than .05 but falls short of corrected significance.

### Computational modeling

Following Fareri, Chang & Delgado (2012), we used a simple RL model in which the expected value *E_a_*(*t*) of each possible action *a* (here, keeping or sharing) is computed as:

\begin{eqnarray*} {E}_{\mathrm{keep}}(t)=1 \end{eqnarray*}

(1) \begin{eqnarray*} {E}_{\mathrm{share}}(t)={Q}_{i}(t)\ast 1.5 \end{eqnarray*}

where $1.5 is the value of the monetary reward that can be obtained from sharing and *Q_i_*(*t*) is the probability of obtaining that reward (i.e., expectation that partner *i* will reciprocate on trial *t*). If the subject decides to keep the money, the subject always gains $1 regardless of partner. Each *Q_i_*(0) is initialized to a value 0 ≤ *θ_i_* ≤ 1, reflecting an estimate of trustworthiness based on that partner’s biography. On subsequent share trials, these estimates are updated based on experience:

\begin{eqnarray*} {Q}_{i}(t)={Q}_{i}(t-1)+{\alpha }_{\mathrm{gain},i}\ast [1-{Q}_{i}(t-1)],\hspace{10.00002pt}\text{if partner ${i}$ reciprocates on trial}t \end{eqnarray*}

(2) \begin{eqnarray*} {Q}_{i}(t)={Q}_{i}(t-1){\alpha }_{\mathrm{loss},i}\ast [0-{Q}_{i}(t-1)],\hspace{10.00002pt}\text{if partner ${i}$ fails to reciprocate on trial}t \end{eqnarray*}

Here, *α*_gain_ and *α*_loss_ are learning rate parameters constrained between 0 and 1 that determine the degree to which expectations *Q_i_* change following a reward (gain) or failure to obtain reward (loss); these parameters are indexed by partner *i* to allow the possibility that these parameters may be different for different partners.

Finally, the probability of deciding to share with partner *i* at time *t* is calculated via a probabilistic response rule:

(3) \begin{eqnarray*} {P r o b}_{\mathrm{share}}(t)=\frac{{e}^{{E}_{\mathrm{share}}(t){}{\beta }_{i}}}{{e}^{{E}_{\mathrm{share}}(t){}{\beta }_{i}}+{e}^{{E}_{\mathrm{keep}}(t){}{\beta }_{i}}}\cdot \end{eqnarray*}

Here, the *β_i_* are “temperature” parameters ranging from 0 to 1 that determine the degree of tendency to repeat previously rewarded interactions with a partner *i* (“exploit”) vs. try alternate actions that might result in greater payoff (“explore”), with higher values of *β_i_* biasing the system to explore and lower values biasing it to exploit.

Thus, there were potentially 12 free parameters in the model: values of *θ_i_*, *α*_gain,*i*_, *α*_*loss*,*i*_, and *β_i_* for each of the three partners *i*. It is important to note that these parameters are common to most reinforcement learning models (e.g., Barto, Sutton & Anderson, 1983; Watkins & Dayan, 1992) and have been implicated in prior simulations of the trust game (Fareri, Chang & Delgado, 2012; Fouragnan et al., 2013). Each parameter was allowed to range from 0 to 1 in increments of 0.01, except as otherwise noted. For each combination of parameter values, for each subject, the model was run on the same trial-by-trial stimulus order as that subject. At each trial, the model action (keep or share) was compared with the subject’s response (keep or share), and the overall fit of the model to subject behavior was assessed by log likelihood estimate across all *n* trials:

(4) \begin{eqnarray*} n e g L L E=-\sum _{t=1}^{n}\ln \hspace{1em}[S h a r e(t)\ast {P r o b}_{\mathrm{ share}}(t)+(1-S h a r e(t))\ast (1-{P r o b}_{\mathrm{share}})]. \end{eqnarray*}

Here, *Share(t)* = 1 if the subject’s action on trial *t* was to share, and *Share(t)* = 0 if the action was to keep. The combination of free parameter values that produced the lowest *negLLE* was defined as the “best-fit” parameter configuration for that subject’s data. We also ran a model that made decisions at “random,” i.e., with a constant *Prob*_share_(*t*) =0.5 for all trials, to ensure that models capable of adjusting responses based on experience with each partner do in fact provide a better fit to the behavior of the participants.

**Table 1:** Reinforcement learning model variations investigated. For all model variations, free parameters were examined in steps of 0.01 except the 5-parameter model (the “3*θ* free *α*, *β*” model), where a step size of 0.05 was used, and the 12-parameter model (the “call free” model) where a step size of 0.2 was used for computational tractability.

Model	Description	Changing parameters	*k*
All free	All 12 parameters free	*β*_Good_, *β*_Bad_, *β*_Neutral_	12 (step size = .2)
		*α*+_Good_, *α*+_Bad_, *α*+_Neutral_	
		*α*−_Good_, *α*−_Bad_, *α*−_Neutral_	
		*θ*_Good_, *θ*_Bad_, *θ*_Neutral_	
3*θ*, fixed *α*, *β*	Fixed *β* = 1, fixed *α* + = *α* − = 0; 3∗*θ* vary	*θ*_Good_, *θ*_Bad_, *θ*_Neutral_	3
3*θ*, free *α*, *β*	Single *β*, single *α* (*α* + = *α* −), 3∗*θ* vary	*β* (*β*_Good=_*β*_Bad=_*β*_Neutral_)	5 (step size =.05)
		*α* + = *α* − (*α*+_Good_ = *α*+_Bad_ = *α*+_Neutral=_*α*−_Good_ = *α*−_Bad_ = *α*−_Neutral_)	
		*θ*_Good_, *θ*_Bad_, *θ*_Neutral_	
3*θ*, free *α*, fixed *β*	Single *α*, fixed *β* = 1, three 3∗*θ* vary	*α* + = *α* − (*α*+_Good_ = *α*+_Bad_ = *α*+_Neutral=_*α*−_Good_ = *α*−_Bad_ = *α*−_Neutral_)	4
		*θ*_Good_, *θ*_Bad_, *θ*_Neutral_	
3*θ*, fixed *α*, free *β*	Fixed *α* = 0 (single *β* varies and 3∗*θ* vary)	*β*(*β*_Good=_*β*_Bad=_*β*_Neutral_)	4
		*θ*_Good_, *θ*_Bad_, *θ*_Neutral_	
LG	Fixed *θ* = 0.5 (single *β*, single *α* +, and single *α* − vary) —the “LG” model of Fareri, Chang & Delgado (2012)	*β* (*β*_Good=_*β*_Bad=_*β*_Neutral_)	3
		*α* + (*α*+_Good_ = *α*+_Bad_ = *α*+_Neutral_)	
		*α* − (*α*−_Good_ = *α*−_Bad_ = *α*−_Neutral_)	

#### Model comparisons

We began by considering the “fully free” model, in which all 12 parameters were allowed to vary (due to computational constraints, parameters in this model were investigated in steps of .2). Our primary hypothesis concerned the effect of initial information, as well as learning rate (represented as *θ* and *α*, respectively). However, we also considered several simpler models where *θ_G_* = *θ_B_* = *θ_N_* but other parameters were fixed or constrained, including a model where learning rates (*α* + and *α* −) and *β* were fixed (at 0, 0 and 1, respectively), a model with only one value of *β* and one learning rate (*α* + = *α* −) for all partners (here, step size = .05), a model where learning rate (*α* + = *α* −) could vary but *β* was fixed at 1, and a model where *β* could vary but learning rate was fixed at *α* + = *α* − = 0. Finally, since Fareri, Chang & Delgado (2012) found that their data were well fit by a model that fixed initial values (all *θ* = 0.5) while allowing one *β*, one *α* +, and one *α* − to vary, we also considered that model. Table 1 summarizes these alternatives. In addition, we considered a number of other models where initial values were fixed and other parameters could vary. However, none of these resulted in a better fit than the models described here and are, therefore, not discussed further.

In general, more complex models that include all free parameters of less complex models should provide as good or better fit to the data (i.e., in terms of *negLLE* obtained under best-fit parameters for each). To balance good fit and model simplicity, Akaike’s Information Criterion (*AIC*) was computed as:

(5) \begin{eqnarray*} A I C=2 k+2 n e g L L E. \end{eqnarray*}

Here, *k* is the number of free parameters in the model (Akaike, 1974). *AIC* penalizes models for number of free parameters in assessing model fit.

#### Data analysis

Model fit was assessed via AIC which takes into account both *negLLE* and the number of free parameters. Since AIC is not necessarily normally distributed, we conducted Friedman’s ANOVA on AIC across different models, with Bonferroni-corrected Wilcoxon’s signed ranks used as post-hoc tests to determine which of the models provided the best, most parsimonious fit. After identifying a best-fitting model, we then conducted analysis of the estimated parameter values as a function of participant gender and BI class (uninhibited or inhibited), to determine if there were group differences in parameter values that might explain how different subgroups of participants approached the task.

## Results

### AMBI

Mean score on the AMBI was 14.7 (*SD* = 5.7); 44 participants (38.6%) were classified as inhibited (AMBI score ≥16), including significantly more females than males (35 of 74 = 47.3% females, 9 of 31 = 29.0% males, Yates-corrected chi-square = 6.74, *df* = 1, *p* = .009). For this reason, and because all partners were male, gender was entered in the analyses of trust game data. However, there was no significant age difference between uninhibited (mean age 21.3y, *SD* = 4.9) and inhibited participants (mean age 21.4y, *SD* = 5.2; *t*(111) = 0.10, *p* = .919).

### Behavioral testing

**Figure 2:**
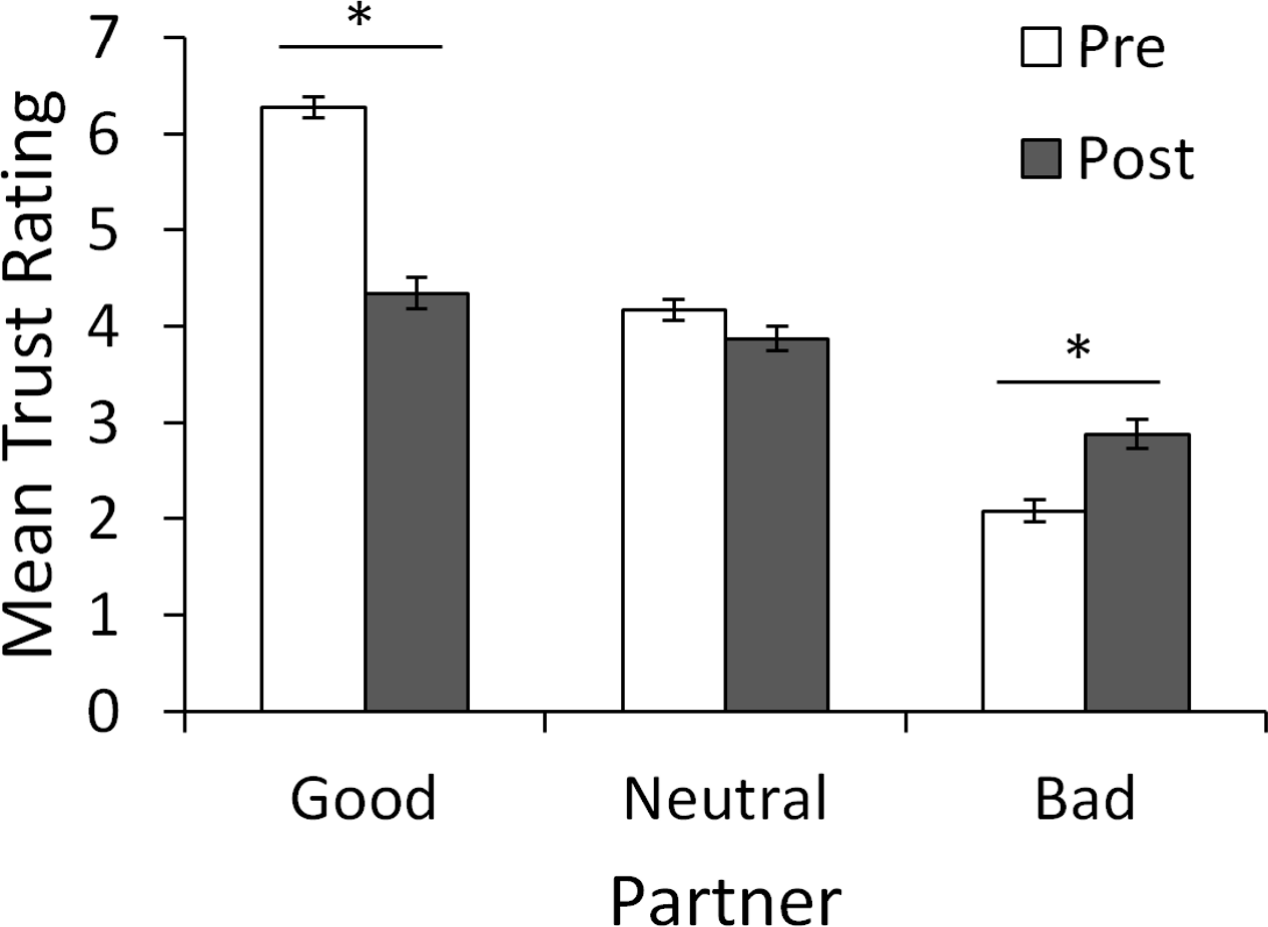
Mean ratings of each partner before and after the trust game. Before the trust game, subjects rated the good partner as significantly more trustworthy, and the bad partner as significantly less trustworthy, than the neutral partner. After the game, ordering was maintained but ratings for the good partner dropped significantly while those for the bad partner increased significantly from pre-game levels. ^∗^ Significant difference between pre- and post-game ratings (*p* < .001).

Multiple linear regression was performed on pre-game trust ratings for the good, neutral or bad partner with score on the AMBI and participant gender entered as predictors. Bonferroni-correction (.05∕3 = .0167) was used to protect significance levels. The regression was not significant for any of the partners (all *p* > .05). The same analysis was also carried out on the change in ratings (computed as post-game minus pre-game ratings) for each of the partners. Once again, the regression was not significant for any of the partners (all *p* > .05). Finally, to ensure our manipulation of trust was successful, we performed repeated-measures ANOVA on trust ratings with factors of partner (good, bad, and neutral) and time (pre- and post-game). Figure. 2 shows initial and post-game ratings for the three partners. Initially (“Pre”), the good partner was rated higher, and the bad partner lower, than the neutral partner; however, after the trust game (“Post”), ratings for all three partners were similar and near the middle of the 7-point range. Repeated-measures ANOVA confirmed these impressions, showing significant main effects of partner, *F*(1.69, 191.28) = 216.561, *p* < .001, ${\eta }_{p}^{2}=.657$, and time, *F*(1, 113) = 27.208, *p* < .001, ${\eta }_{p}^{2}=.194$, and an interaction between partner and time, *F*(1.89, 213.40) = 70.058, *p* < .001, ${\eta }_{p}^{2}=.383$. Bonferroni-corrected (.05∕9 = .0056) post-hoc pairwise *t*-tests confirmed that, before testing, ratings were significantly higher for the good partner compared to the neutral, *t*(113) = 14.63, *p* < .001, or bad partner, *t*(113) = 24.88, *p* < .001, and significantly higher for the neutral than the bad partner, *t*(113) = 13.84, *p* < .001; this ordering was maintained after testing (all *p* < .001 except good vs. neural, *p* = .007). Comparing pre-post ratings, there was a significant drop in ratings for the good partner, *t*(113) = 11.02, *p* < .001, and a significant increase in ratings for the bad partner, *t*(113) = 4.80, *p* < .001. However, the change in ratings of the neutral partner fell short of corrected significance, *t*(113) = 2.08, *p* = .039.

In summary, the biographies were effective in manipulating initial ratings, and ratings changed as a result of the trust game, but there was no obvious effect of BI or gender.

**Figure 3:**
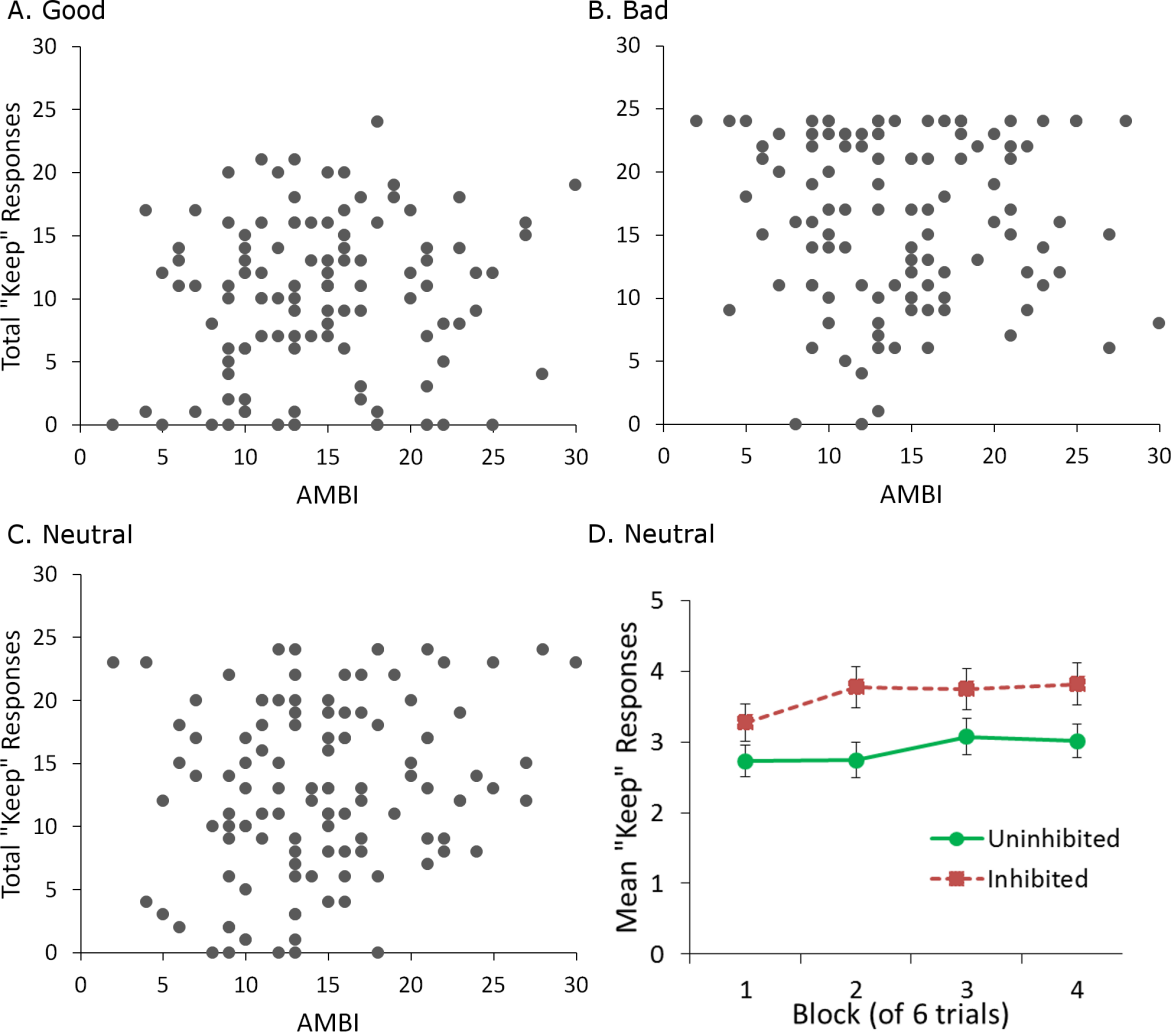
Behavioral choices in the trust game. (A–C) Higher score on the AMBI, indicating greater BI, was associated with significantly more “keep” responses in the trust game, but only for the neutral partner. No relationship between AMBI score and “keep” responses was found for the good and bad partners. (D) Mean “keep” responses per block (of 6 trials) for the neutral partner, for subjects classified as uninhibited (AMBI < 16) or inhibited (AMBI ≥16). Inhibited participants made significantly more “keep” responses than uninhibited participants when dealing with the neutral partner but there was no effect of block and no interaction of block with BI. Error bars represent SEM.

Multiple linear regression was also performed on total “keep” responses for each partner (good, neutral or bad) during the trust game, with score on the AMBI, participant gender, and pre-game ratings for the corresponding partner entered as predictors. Bonferroni-correction (.05∕3 = .0167) was used to protect significance levels. The regression was significant only for the neutral partner (*R*^2^ = .344, *F*(3, 110) = 4.934, *p* = .003). Score on the AMBI significantly predicted total “keep” responses (*β* = .279, *p* = .012), where participants with higher AMBI scores tended to keep more when dealing with the neutral partner (Fig. 3C). Pre-game ratings were also a significant predictor (*β* = − 1.517, *p* = .005), with lower ratings associated with more keep responses. The regression failed to reach significance for both the good and bad partners (both *p* > .05, Figs. 3A–3B).

Taken together, these results show that self-reported ratings of trustworthiness and behavior in the trust game were dissociated for inhibited participants when dealing with the neutral partner. While AMBI score failed to predict ratings assigned to the neutral partner, higher AMBI score was associated with lower trust in the game. As trustworthiness was reported only before and after, this discrepancy could be due to differences in how inhibited participants learned throughout the game. That is, inhibited participants may have initially kept as much as uninhibited participants, increased how much they kept later on, and then decreased how much they kept by the end of the game, accounting for the similar ratings of the neutral partner before and after the game. To examine this possibility, we performed a mixed-model analysis of covariance on “keep” responses for the neutral partner, with factors of block and BI, and a covariate of pre-game rating for the neutral partner. A block consisted of 6 trials with that partner. For this analysis, participants with a score less than 16 on the AMBI were classed as “uninhibited,” with the remainder classed as “inhibited.” As described earlier, this cutoff represents the median for the scale, and is also close to the median (equal to 14) of the current sample. Gender was not included as a factor since the regression did not identify it as a significant predictor of “keep” responses. This analysis revealed only significant main effects of pre-game rating, *F*(1, 111) = 7.157, *p* = .009, ${\eta }_{p}^{2}=.061$, and BI, *F*(1, 111) = 5.049, *p* = .027, ${\eta }_{p}^{2}=0.44$ (Fig. 3D). There were no interactions, or a main effect of block (all *p* > .05). Thus, inhibited participants simply appear to keep more overall when dealing with the neutral partner, suggesting that the dissociation between “keep” responses and trust ratings is maintained throughout the game.

Finally, to ensure that our manipulation of trust was successful, we performed repeated-measures ANOVA on total “keep” responses for each partner (good, bad, and neutral). This confirmed a significant effect of partner, *F*(1.86, 210.17) = 32.521, *p* < .001, ${\eta }_{p}^{2}=.223$. Bonferroni-corrected (.05∕3 = .0167) post-hoc pairwise *t*-tests confirmed that participants made significantly more “keep” responses, indicating lower trust, when dealing with the bad partner compared to both the neutral partner, *t*(113) = 4.693, *p* < .001, and the good partner, *t*(113) = 7.136, *p* < .001. Finally, they also made more “keep” responses to the neutral partner compared to the good partner, *t*(113) = 3.978, *p* < .001, confirming that the biographies were successful in manipulating the trustworthiness of the partners.

**Figure 4:**
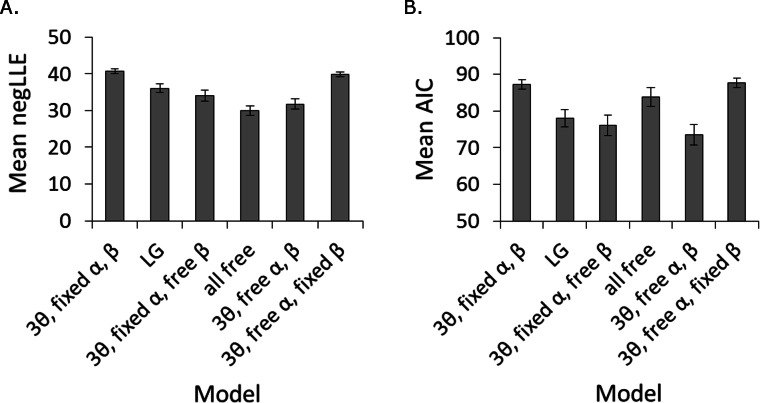
Success of different models in fitting participant data. Model fit was assessed in terms of (A) *negLLE*, and (B) *AIC*, which takes into account model complexity (number of free parameters). The “3*θ*, free *α*, *β*” and “3*θ*, fixed *α*, free *β*” models were statistically tied for best fit in terms of *AIC*. The rest of the analysis focuses on the “3*θ*, free *α*, *β*” model since it has numerically lower *AIC* and has a free learning rate parameter (*α*), which was predicted to differ between inhibited and uninhibited individuals. Note that since each model was fit to every participant, there were a total of 114 simulations per model (the same as the total number of participants). Error bars represent SEM.

### Computational modeling

Figure 4 shows how well the different models fit the behavioral data, in terms of both *negLLE*, and *AIC*, averaged across subjects. Note that the “random” model (not shown) produced an average *negLLE* = 49.8 (SD 3.5) for this dataset, and therefore provided the worst fit to the data. As in Fareri, Chang & Delgado (2012), this indicated participants did make decisions based on experience with the partners throughout the game. Friedman’s ANOVA on AIC confirmed there were significant differences among the remaining models, *χ*^2^(5) = 138.536, *p* < .001, and Wilcoxon signed ranks post-hoc pairwise comparisons using Bonferroni correction (alpha adjusted to .05∕15 = .0033) confirmed that the “LG,” the “3*θ*, fixed *α*, free *β*,” and the “3*θ*, free *α*, *β*” models all had significantly lower *AIC* than the remaining models, including the “all free” model. The “LG” and “3*θ*, fixed *α*, free *β*” models were not significantly different from each other, *z* = − .406, *p* = .685, *r* = − .03, but the “3*θ*, free *α*, *β*” model had significantly lower *AIC* than the “LG” model, *z* = − 4.429, *p* < .001, *r* = − .29. Finally, the difference between the “3*θ*, free *α*, *β*” and “3*θ*, fixed *α*, free *β*” models failed to reach corrected significance, *z* = − 2.383, *p* = .017, *r* = − .16. These models were, therefore, statistically tied for best fit.

**Figure 5:**
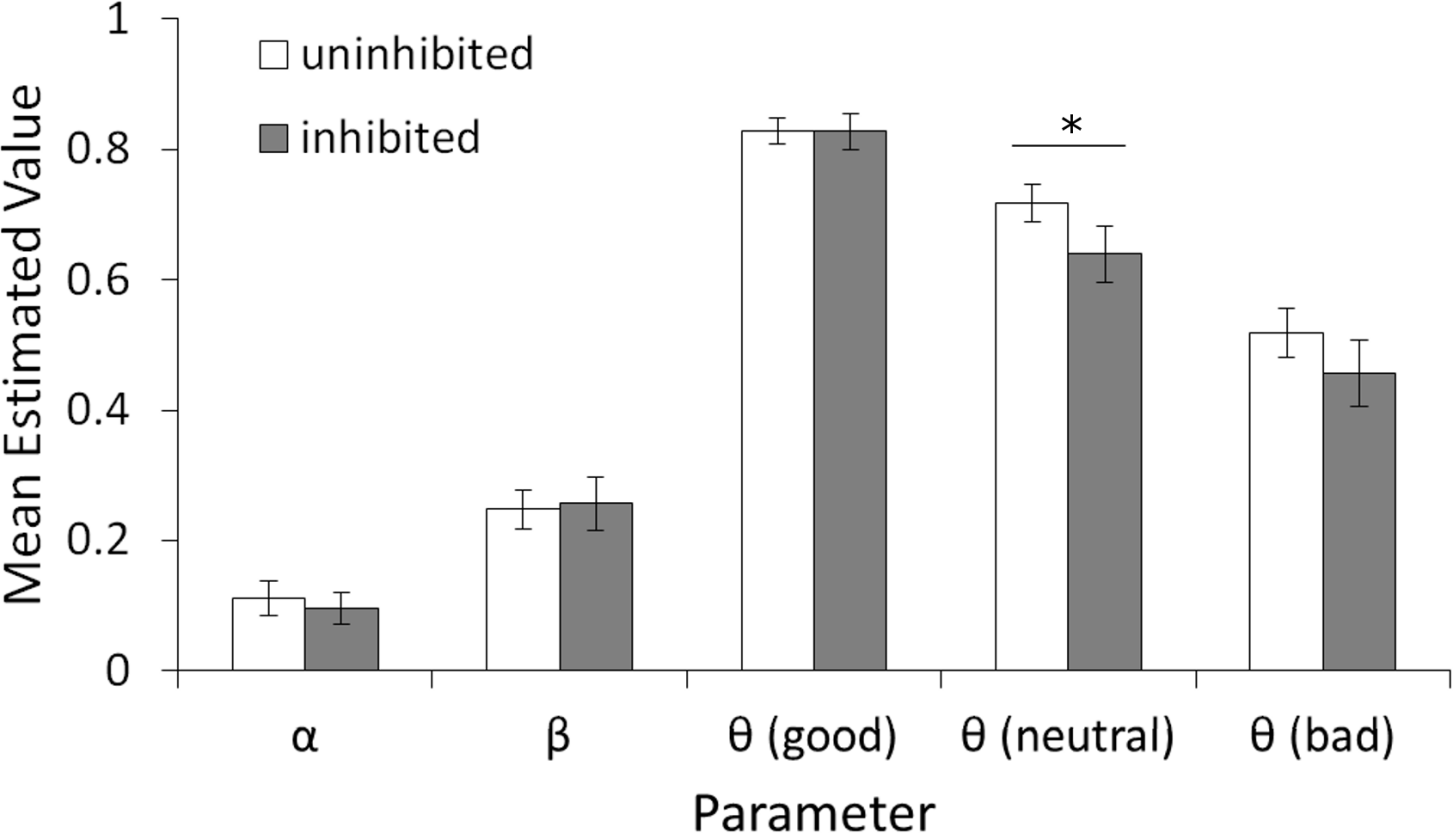
Values of estimated parameters generated by the 3*θ*, free *α*, *β* model split by participant BI. (A) Mean estimated values of the learning rate (*α*), temperature parameter (*β*), and *θ*_Good_, *θ*_Bad_, and *θ*_Neutral_ representing initial belief that each partner will reciprocate. For *α* and *β*, no significant main effects or interactions with BI or gender (not shown) were found. All three *θ* parameters were significantly different from each other (good > neutral > bad, all *p* < .001). The main effect of BI reached corrected significance only for the value *θ*_Neutral_ associated with the neutral partner (*p* = .009). Error bars represent SEM.

Given that the only difference between the two models is whether the learning rate parameter (*α*) was fixed or allowed to vary, and we had initially predicted that learning rates may differ between inhibited and uninhibited individuals, we selected the “3*θ*, free *α*, *β*” model as the best fit model and focus on it for the rest of the analysis. Despite having an additional free parameter, it still had the lowest *AIC* (numerically) and contained all parameters of interest, including three initial values *θ_i_* corresponding to initial trust given to each of the three partners, one learning rate *α*, and one temperature *β*. Figure 5 shows estimated parameter values from this model in participants classed as inhibited vs. uninhibited. Univariate ANOVAs on *β* and *α* confirmed no effect of gender or BI and no interaction (all *F* < 1, all *p* > .600). Mixed-model ANOVA on the three *θ* values, with between-subjects factors of participant BI (inhibited or uninhibited) and gender, confirmed a main effect of partner, *F*(1.84, 202.71) = 47.863, *p* < .001, ${\eta }_{p}^{2}=.303$, with estimated values for *θ*_Good_ larger than for *θ*_Neutral_, *t*(113) = 5.51, *p* < .001, *r*^2^ = .21, and values for both *θ*_Good_ and *θ*_Neutral_ larger than values for *θ*_Bad_, *t*(113) = 10.22, *p* < .001, *r*^2^ = .48, and *t*(113) = 6.27, *p* < .001, *r*^2^ = .26, respectively. There was also a significant effect of BI, *F*(1, 110) = 6.728, *p* = .011, ${\eta }_{p}^{2}=.058$, and a gender-BI interaction, *F*(1, 110) = 7.261, *p* = .008, ${\eta }_{p}^{2}=.062$, although the main effect of gender fell short of significance, *F*(1, 110) = 3.683, *p* = .058, ${\eta }_{p}^{2}=.032$. To further investigate the interaction, we conducted follow-up ANOVAs on the three *θ* values with Bonferroni correction (alpha adjusted to .05∕3 = .0167) to protect significance. For both *θ*_Good_ and *θ*_Bad_, the interaction between gender and BI approached uncorrected significance (.05 < *p* < .1); for *θ*_Neutral_, there was a main effect of BI, *F*(1, 110) = 7.104, *p* = .009, ${\eta }_{p}^{2}=.061$, while both the main effect of gender, *F*(1, 110) = 3.629, *p* = .059, ${\eta }_{p}^{2}=.032$, and the gender-BI interaction, *F*(1, 110) = 5.497, *p* = .021, ${\eta }_{p}^{2}=.048$, did not reach corrected significance. Thus, *θ*_Neutral_ was the only parameter that varied as a function of BI, indicating significantly higher estimates of trustworthiness for the neutral partner in uninhibited than inhibited participants. This result is consistent with the observed dissociation between declarative ratings and “keep” responses for the neutral partner in the trust game, increasing our confidence in this model.

## Discussion

The current study employed the trust game to examine whether individuals who score high on the personality trait of BI differ in how they make decisions based on perceptions of moral character. Self-reported ratings of trust confirmed that all participants, irrespective of BI, perceived differences in the moral character of the three partners (trustworthiness of good > neutral > bad partner). The results also show that inhibited participants tended to trust the neutral partner less than uninhibited participants (i.e., inhibited participants chose to keep significantly more when dealing with the neutral partner) despite receiving identical feedback. In contrast, this was not reflected in the ratings of the neutral partner (either pre- or post-game), which were similar for both inhibited and uninhibited participants. Thus, self-reported ratings of trust by inhibited participants were dissociated from the decisions they made in the trust game.

Computational modeling indicated that differences between inhibited and uninhibited participants in either the learning rate or the tendency to explore new responses (e.g., share instead of keep in the game) vs. repeat previously made responses (e.g., continue to keep as opposed to share), could not account for this pattern. Rather, according to the model, inhibited participants started the game with a lower estimate of trustworthiness for the neutral partner. Taken together with the experimental data, these results indicate that inhibited individuals tend to treat ambiguous conditions more negatively and that this bias is implicit, i.e., not available declaratively. These results build on previous studies that have shown decisions to trust can rely on implicit biases (Delgado, Frank & Phelps, 2005; Chang et al., 2010; Stanley et al., 2011; Fouragnan et al., 2013), direct social experience (Fareri, Chang & Delgado, 2012) and biographical information about moral character (Delgado, Frank & Phelps, 2005). For example, Delgado, Frank & Phelps (2005) also found that declarative ratings were dissociated from behavior in the trust game. That is, participants tended to continue sharing with the good partner, despite receiving feedback that was inconsistent with their expectations (i.e., the partner did not reciprocate as much as expected). Behaviorally, participants tended to ignore the feedback. In contrast, declarative trust ratings did change—from pre- to post-game, ratings tended to move toward neutral. Similarly, Fouragnan et al. (2013) reported that when prior information was provided, participants tended to rely on this information despite inconsistent behavior by the partner. In the current study, inhibited participants tended to rate the neutral partner as neutral, but still chose to keep more when playing with that partner during the game. Therefore, declarative ratings of trust and decisions in the trust game were dissociated. These participants treated the neutral partner as less trustworthy in the game. In contrast, participants with lower BI both rated and treated the neutral partner as neutral.

Fareri, Chang & Delgado (2012) also applied computational modeling to understand behavior in the trust game. Their best fit model corresponded to the “LG” model in the current study, and suggested there was a confirmation bias such that participants updated their beliefs faster when behavior of the partners matched expectations, i.e., when the good partner shared and the bad partner refused to share. Similar evidence of a confirmation bias was obtained by Doll et al. (2009), where modeling suggested that subjects tended to discount outcomes that were inconsistent with expectations more than they amplified those that were congruent. Here, however, the best fit model had identical learning rates from gains and losses (*α* + = *α* −) irrespective of partner, precluding observing a confirmation bias. The best-fit model of Fareri, Chang & Delgado (2012) also employed a single initial value (*θ*), which was set to 0.5 irrespective of partner. Here, the best-fit model had three separate values of *θ* (one for each partner), which were allowed to vary. This was similar to Fouragnan et al. (2013), who selected a model with separate initial values for each of the two partners in their study.

Besides sampling error, differences in how trustworthiness was manipulated could account for the discrepancy between the models: Fareri, Chang & Delgado (2012) used a ball game, Fouragnan et al. (2013) provided information about how the partners behave in similar types of games (i.e., how much they value their own profit vs. that of others), while the current study manipulated trustworthiness through biographical information, similar to Delgado, Frank & Phelps (2005). However, several other methodological differences make direct comparisons to Fouragnan et al. (2013) difficult. It is also important to note that there were two best-fit models to choose from in the current study, and the “LG” model selected by Fareri, Chang & Delgado (2012) provided the next best fit. Given our hypotheses about the role of BI, we were specifically interested in a model that allowed for differences in trust between the partners, as well as learning rate, which guided our selection. This selection was also justified by the behavior of inhibited participants in the trust game, which indicated they were specifically less trusting of the neutral partner. Nonetheless, the model we selected could be considered preliminary, and should be tested on different data sets collected from future studies that examine the role of BI, or other personality traits, in social trust.

To our knowledge this is the second study to examine the relationship between BI and trust (see Oskarsson et al., 2012), and the first to do so through a behavioral measure of trust. Oskarsson et al. (2012) found higher scores on the AMBI for both males and females to be associated with lower social trust. Trust was measured based on response to the question “Generally speaking, would you say that most people can be trusted, or that you can’t be too careful in dealing with people?”, which was scored on a 10-point response scale (Oskarsson et al., 2012, p. 23). The current study shows inhibited individuals are less trusting, specifically when information about the social partner is neutral or ambiguous. The results also highlight the importance of including behavior to complement self-report measures in future studies of the relationship between BI and trust, as the two may not always agree. To avoid confusion, it is important to note that Oskarsson et al. (2012) took the reverse of the AMBI scores as a measure of extraversion, a personality trait associated with sociability, eagerness to engage with others and develop new social relationships. Indeed, there is a high negative correlation between the AMBI and standard measures of extraversion (Gladstone & Parker, 2005) and prior studies have found high extraversion to be associated with greater social trust (e.g., Hiraishi et al., 2008). On the other hand, BI is also related to neuroticism (Shatz, 2005). Future studies could confirm whether similar results are found using alternative measures of BI, such as the Behavioral Inhibition Scale (Shatz, 2005).

Another important direction for future research will be to examine how BI modulates the relative influence of the neural substrates involved in perceived trustworthiness and decision making. Functional imagining studies on the trust game (e.g., Delgado, Frank & Phelps, 2005; Fouragnan et al., 2013) have found that when there is no prior information (i.e., lottery trials with a random outcome), or there is neutral information (i.e., biography of the neutral partner), decisions appear to rely more on the caudate nucleus. That is, there is differential activation in the caudate on lottery trials, trials involving the neutral partner, and to a lesser extent the bad partner, depending on the type of decision (share or keep) and the outcome (positive or negative, but see Fareri, Chang & Delgado, 2012). In contrast, decisions involving the good partner appeared to rely more on other structures, including the insula and cingulate cortex (Delgado, Frank & Phelps, 2005). It remains unknown how BI is related to the involvement of these areas in social trust. The current study suggests that decisions to trust the neutral partner were mediated by an implicit bias as a function of BI. Therefore, the hippocampus might be differentially involved in inhibited individuals. Other imaging studies have shown that responses of the hippocampus and amygdala of inhibited individuals fail to habituate when viewing neutral faces (Blackford et al., 2012) and higher BI in childhood is associated with smaller hippocampal volume in adolescence (Schwartz et al., 2015). Specific regions of the cerebellum of inhibited individuals also show greater connectivity to the dorsolateral prefrontal cortex (Caulfield et al., 2015)—an area involved in executive control that has also been implicated in encoding past decisions and their outcomes, as well as influencing future decisions, in other types of socioeconomic games (Rilling, King-Casas & Sanfey, 2008). Future studies could examine the relative involvement of different neural networks in inhibited relative to uninhibited individuals during the trust game, and whether this depends on the type of partner, as well as decision outcome.

BI is also a risk factor for anxiety disorders (Rosenbaum et al., 1993; Turner, Beidel & Wolff, 1996; Mick & Telch, 1998; Clauss & Blackford, 2012; Clauss, Avery & Blackford, 2015) and AMBI scores are higher in individuals with severe symptoms of post-traumatic stress disorder (PTSD, Myers et al., 2013; Myers, VanMeenen & Servatius, 2012). Several studies have also documented enhanced associative learning by BI individuals in domains such as eyeblink classical conditioning (Caulfield, McAuley & Servatius, 2013; Holloway et al., 2014) and computer-based operant tasks (Sheynin et al., 2013; Sheynin et al., 2014). As such, we expected there might also be enhanced learning by participants with high BI scores on the current task. However, we found no relationship between BI and any behavioral measure, or estimated model parameters, in the current study. Future studies could examine more closely what features of learning tasks produce facilitated learning in inhibited individuals. It is possible that enhanced associative learning is observed only for non-social stimuli, and only under certain conditions. For example, faster delay eyeblink conditioning in inhibited adolescents was reported for short (500 ms), but not long (1,000 ms) delays (Caulfield, VanMeenen & Servatius, 2015).

A limitation of the current study is that the partners were always white and male. While these images were intentionally selected to replicate the prior Delgado, Frank & Phelps (2005) study, this did limit exploration of what effects partner appearance might have on participant behavior and trust ratings. While prior studies have found mixed results, in general, perceptions of trustworthiness and the actual willingness to trust others can depend a number of factors, including gender (Buchan, Croson & Solnick, 2008) and implicit race attitude (Stanley et al., 2011). For example, in a trust game involving a single interaction, males tended to send more money while females tended to return more. However, this did not depend on the gender of the partner (Buchan, Croson & Solnick, 2008). In a prisoner’s dilemma, both men and women stated women were more likely to cooperate but this belief was not expressed in behavior. That is, women were rated as more trustworthy but neither male or female participants chose to play with women more often than with men (Orbell, Dawes & Schwartz-Shea, 1994). Stanley et al. (2011) found that those with pro-white implicit racial bias tended to find white partners more trustworthy while those with a pro-black implicit bias tended to find black partners more trustworthy. Finally, the ability to select a partner may amplify gender or other biases in trust. For example, Slonim & Guillen (2010) found that given the ability to select a partner, participants tended to select partners of the opposite gender, and that males tended to send more to female partners only when they were allowed to choose that partner. We did not find significant gender effects or interactions between gender and BI. However, in the current sample, inhibited males were underrepresented and therefore the study may have lacked the power to detect these effects. Future studies could examine the relationship between BI and trust when partners are of the same vs. different gender or race, as well as when participants are allowed to choose their partner vs. have it assigned to them, and with a larger sample of inhibited males.

It is also worth noting that as in other studies (Delgado, Frank & Phelps, 2005; Fareri, Chang & Delgado, 2012; Fouragnan et al., 2013), partners in the current study were controlled by the computer. While participants were not explicitly told the partners were fictional, most, if not all, participants were likely aware of this fact. Future work could test whether the results differ when the partners are human (e.g., located in the same room) as in other studies with similar games (e.g., Orbell, Dawes & Schwartz-Shea, 1994).

## Conclusion

Our initial hypothesis was partially supported—while inhibited participants were less trusting than uninhibited participants, this was limited to dealing with the neutral partner and suggested a tendency to interpret neutral or ambiguous information more negatively. However, we found no evidence that inhibited participants adjusted their beliefs faster than uninhibited participants. Surprisingly, inhibited participants also rated all partners similar to uninhibited participants. Computational modeling indicated that the bias shown by inhibited participants was due to lower initial trust of the neutral partner rather than a higher learning rate associated with loss. This bias appears to be implicit given that initial ratings of trustworthiness for the neutral partner were identical for both inhibited and uninhibited participants. The results also suggest that inhibited individuals may be rated as less trustworthy by others in real-life social interactions given they are less willing to trust, in particular when only limited information is available. This could have important negative implications for the ability of inhibited individuals to engage in social relationships, which could in turn feed into the tendency to avoid unfamiliar people characteristic of behaviorally inhibited temperament, as well as its relationship to anxiety disorders.

## Supplemental Information

10.7717/peerj.1631/supp-1 Supplemental Information 1 Raw data files from the trust game Text files containing data from each participant in the trust game. A separate line for each trial in the game indicates the type of partner (good, neutral or bad) for that trial, the face corresponding to the partner, whether the participant shared, and if so, whether the partner also shared.

10.7717/peerj.1631/supp-2 Supplemental Information 2 Participant demographics and questionnaire scores Gender, age and score on the adult measure of behavioral inhibition for each participant with a summary of corresponding partner ratings and behavior in the trust game.

10.7717/peerj.1631/supp-3 Supplemental Information 3 Computational modeling data The first tab contains LLE and AIC for each model. Parameter values for the best fit model are shown on the second tab.

## Appendix. Partner Biographies

Partner biographies were modified (shortened and simplified) from those used in Delgado, Frank & Phelps (2005), and presented together with partner name and photo. Assignment of names and pictures to biographies was counterbalanced across subjects. Pictures were the same as those in the prior paper, and were from the NimStim Face Stimulus Set (Tottenham et al., 2002; Tottenham et al., 2009). The biographies are shown below, with one possible partner name filled in.

### Good partner

Alex Thompson works as a high school English teacher in Camden, NJ. Last year, Thompson was at a movie with his niece Susan when a fire broke out in the darkened theater. Nineteen people were trapped inside and died. Thompson had been seated near the exit, and was able to lead his niece and several others out of the theater. At this point, the firemen had not yet arrived. Thompson went back inside the smoke-filled theater and found a woman lying unconscious on the floor overcome by smoke. He managed to drag her to safety. In the process, he suffered third degree burns to his neck, chest and both arms. Thompson spent three weeks in the hospital recovering before being released.

### Bad partner

*Christopher Zizzo works as a financial analyst in New York City. Earlier this year, Zizzo was arrested on charges of attempting to sell counterfeit handicapped parking stickers to three men whom he believed to be Manhattan motorists, but who in fact were undercover New York City police officers. Police said the arrest was part of an ongoing operation targeting parking fraud in the city. During his arraignment, Zizzo admitted to making more than* $*5,000 selling the fake stickers. Zizzo said later that he didn’t think his actions caused any real harm to anyone, because there are far more handicapped spaces in city parking facilities than are really necessary. Zizzo was released on bail, pending a trial scheduled for early next year*.

### Neutral partner

James Sweeney works as a mechanical engineer for a manufacturing firm in Bayonne, NJ. According to the local newspaper, Sweeney narrowly missed death last year when a US Airways Express flight crashed in a fiery explosion shortly after takeoff, killing all 21 persons on board. Sweeney had booked a seat as a passenger on the flight, but because of unusually heavy traffic on the highway, Sweeney arrived at the airport an hour later than he’d planned, and discovered that the plane had pushed back from the gate just seconds earlier. In an interview for the newspaper, Sweeney said he had been shaken by the close call, and expressed sympathy for the families of victims, but added, “You can’t control fate.”

## Additional Information and Declarations

### Data Availability

The following information was supplied regarding data availability: The raw data set has been uploaded as supplemental files.
